# Experimental Study on Coupling Influence of Temperature and Confining Pressure to Deformation and Strength Characteristics of Rock-like Material with Pre-Existing Crack

**DOI:** 10.3390/ma14247572

**Published:** 2021-12-09

**Authors:** Hongwei Wang, Yongyan Wang, Xi Fu

**Affiliations:** 1 College of Mechatronics and Vehicle Engineering, Weifang University, Weifang 261061, China; fuxigo@wfu.edu.cn; 2 College of Electromechanical Engineering, Qingdao University of Science and Technology, Qingdao 266061, China; wangyongyan168@163.com

**Keywords:** strength characteristic, peak stress, temperature, confining pressure, initial crack

## Abstract

In this paper, destructive compression tests under the coupled influence of temperatures (20–60 °C) and confining pressures (0–7 MPa) were carried out on rock-like material with pre-existing crack to explore the deformation and strength characteristics. The stress–strain curves of rock-like material under the coupled influence of temperatures and confining pressures were obtained. Meanwhile, the correlations of peak stress, peak strain, and average Young’s modulus with temperatures and confining pressures were obtained. The results of the experiments indicate that, firstly, the compressive strength decreased and the deformation increased due to the influence of pre-existing cracks; the combined effect of initial cracks, temperature, and confining pressure gave rise to a more complicated mechanism of soft rock deformation. Secondly, the deformation of rock-like material was affected by initial cracks, confining pressures, and temperatures, but the influence of temperature was lower than that of confining pressure and initial crack. The failure mode of rock-like material was brittle at the confining pressure of 0 and 1 MPa and plastic at the confining pressure of 5 and 7 MPa. The critical confining pressure value of failure mode for rock-like material was 3 MPa. Thirdly, the peak strength and peak strain of rock-like material increased with confining pressure. Temperature had less influence on the rock-like material strength and peak strain than confining pressure. Lastly, Young’s modulus decreased with temperature and confining pressure.

## 1. Introduction

With the rapid development of the economy, the need for energy is growing fast. Meanwhile, production from shallow resources is running out, and the exploitation of deeper energy resources is increasing. Recently, the maximum mining depth reached 1500 m in China and the maximum mining depth of other countries also reached over 1000 m. The mechanical properties of deep rock mass are different from shallow rock mass. Three characteristics of the deep environment are high ground temperature, high ground stress, and high hydraulic pressure [1]. The mechanical properties of deep rock mass under the influence of high temperature and high stress are greatly different from those of shallow rock mass [2]. There are several cracks in deep rock mass [3], and these discontinuous flaws have a great influence on the mechanical properties [4]. Numerous scholars have devoted their efforts to the study of rock mechanical properties under different temperatures and confining pressures. For example, the influence of confining pressure on the deformation and strength characteristics of ice-rich sandstone depends on the content of ice [5]; the failure mode of marble transforms from brittle failure to plastic failure with the increase in confining pressure [6]; volume expansion was found under the effect of confining pressure, and the Young’s modulus of shale increased with confining pressure at certain temperatures [7]. Temperature also has significant influence on the mechanical properties; the peak stress of specimens decreased rapidly with temperature from 25 to 85 °C, whereas it decreased gradually with temperature from 85 to 135 °C [8]. Burghignoli found that a change in temperature from 20 to 60 °C was insignificant toward the deformation and strength characteristics of clay [9]. The failure mode of kaolin transformed from brittle failure to plastic failure at 90 °C, and the shear strength of the specimen increased simultaneously [10]. In Masri’s research, both confining pressure and temperature had a significant influence on shale strength, which decreased with temperature and increased with confining pressure [11]. Xu studied the strength characteristics of granite after high-temperature treatment, revealing that both peak strength and shear strength increased with confining pressure, whereas cohesion decreased linearly with temperature [12]. Zhou found that the influence of temperature on sandstone strength was related to confining pressure [13]. Fang investigated the dynamic strength characteristic of halite under the effect of confining pressure and temperature, showing that the dynamic peak stress of halite decreased with temperature, whereas the confining pressure had a significant effect on the ductility of halite [14]. Rossi found that rock strength decreased with temperature [15]. Furthermore, Huang investigated the influence of crack angle on rock strength, which showed that peak stress and Young’s modulus decreased first and then increased with fissure angle, whereas peak strain increased with fissure angle [16]. Zhou found that both the peak stress and the peak strain decreased with fissure angle [17]. Liu investigated the influence of single fissure on the fracture mode of a specimen [18]. Han et al. [19] and Zhang et al. [20] investigated the influence of stress path and temperature stress on the strength characteristics of an initial fissure specimen, respectively. Huang [21] and Wang [22] investigated the influence of pre-existing fissure angle on the rock-like material strength, and they found that the maximum fissure angle at which confining pressure exhibited the greatest influence on rock-like material strength was 45°. Zeinab [23] investigated rock masses containing various discontinuities with 3D printing technology, demonstrating that the bonded particle model is capable of precisely modeling the crack initiation location and type, as well as the coalescence type. Lin [24] investigated jointed rock-like specimens with two dissimilar layers, showing that peak strength is associated with the joint angle and the rock bridge angle.

The above investigations focused on one or two factors of temperature and confining pressure, and the specimens used in their investigation were intact. However, there several fissures and cracks in rock mass. Thus, it is necessary take the initial fissures and cracks into consideration during investigation. Until now, no research considered the combined influence of temperature and confining pressure on rock mass with fissure and cracks. Hence, this paper set out to investigate the influence of temperature and confining pressure on the strength and deformation characteristics of specimens with initial cracks, so that the combined influence of temperature, confining pressure, and initial fissure could be obtained. The specimen we used in this paper was rock-like material, and the initial fissure angle was 45°. We investigated the strength characteristics of the rock-like material by compression test under different temperatures and confining pressures. This study is of great significance for clarifying the deformation law and failure mechanism of deep damaged rock mass, as well as ensuring the safety and efficient development of deep geotechnical engineering.

## 2. Rock-like Material and Testing Procedure

### 2.1. Rock-like Material

**Figure 1:**
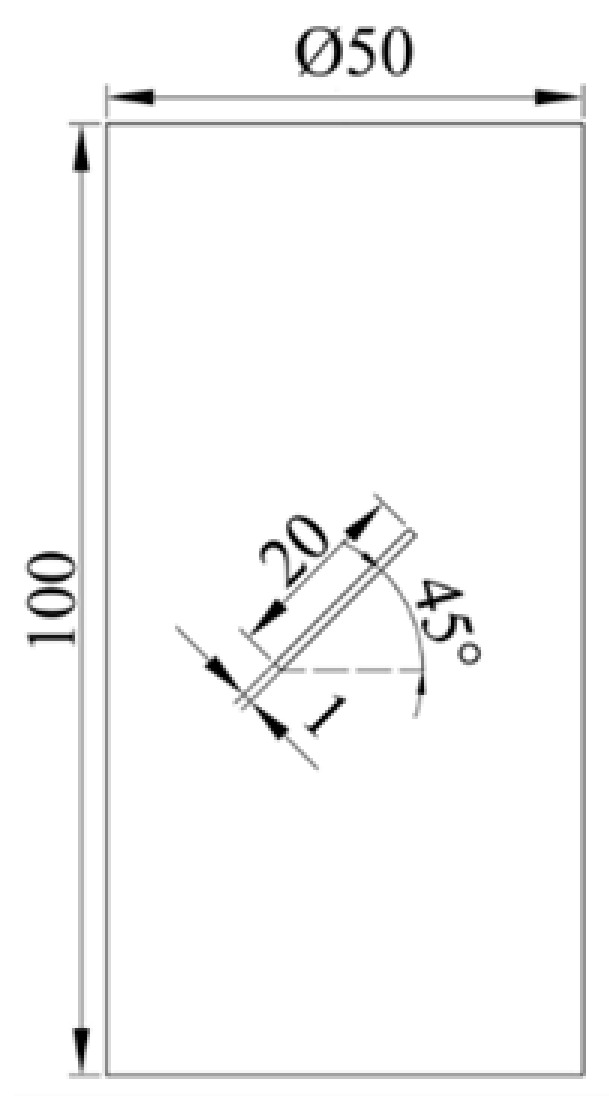
Diagram of shale-like specimen.

**Figure 2:**
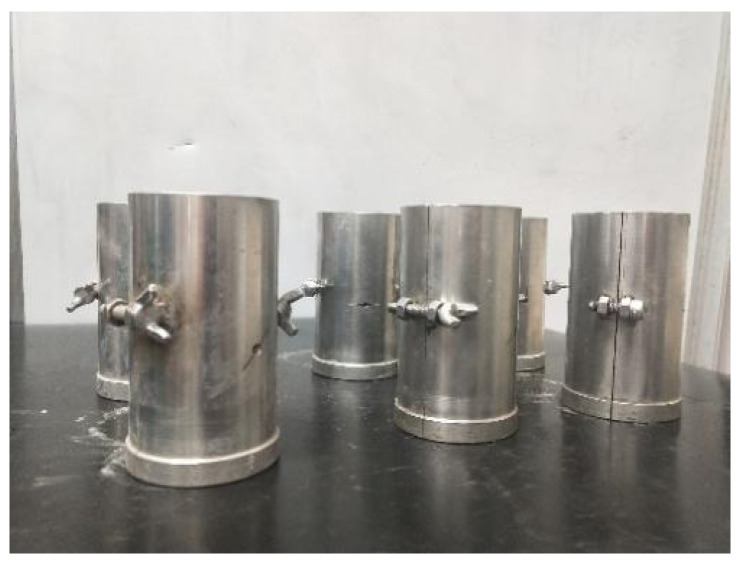
Specimen mold.

Considering the difficulty of making regular cracks on real rock mass, and that it would change the mechanical properties, the specimens used in this paper were rock-like material with similar mechanical properties to shale, whereby regular cracks could be made easily. The components of the rock-like material were river sand, cement, gypsum powder, and water (2:1:0.36:0.36) [25,26]. The size of the standard test specimen was 50 mm in diameter and 100 mm in length, which follows the Chinese standard of *Standard for test methods of engineering rock mass* [27]. The end-face non-parallelism error of the specimen was less than 0.05 mm, the diameter error was less than 0.3 mm, and the deviation of the end face from the axis of the specimen was less than 0.25°. A diagram of the specimen model is shown in Figure 1, and the specimen mold is shown in Figure 2.

**Figure 3:**
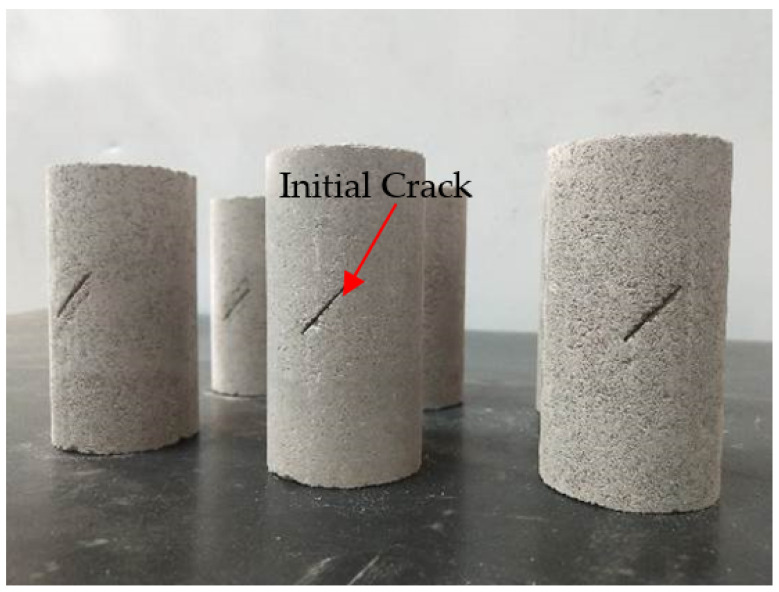
Prepared specimens of rock-like material with initial crack.

Furthermore, we found that the initial cracks would decrease the strength of the rock-like material, while the range of the decreasing amplitude depended on the fissure angle in a previous study. We found that the minimum strength always occurred when the fissure angle was 45°, which means that a 45° fissure angle had the most significant influence on the mechanical properties of the rock-like material. Thus, we chose 45° as the angle of initial fissure in this paper to exhibit the most significant difference in mechanical properties and deformation between soft rock and rock-like material. The initial fissure was located in the middle of the specimen, and the fissure angle was 45° along the horizontal plane. The size parameters of the fissure were 20 mm in length, 1 mm in thickness, and 15 mm in width, as shown in Figure 1. The samples were made followed the method described in [23]. The pre-existing crack is made using a steel sheet with the same dimensions of the cracks. First, we inserted the steel sheet into the specimen with the specimen compressed, before removing it upon achieving the desired shape. The prepared specimens are shown in Figure 3. The specimens were compacted in a specimen mold, and then all specimens are molded for 2 h to ensure the integrity of the fissure. All specimens were air-dried for 28 days in a ventilated place before the compression test, ensuring that all samples had similar strength.

Before the formal investigation, we carried out a serious of tests on rock-like material, and the parameters are shown in Table 1. We can see from Table 1 that the parameters of the rock-like material were similar to those soft rock, suggesting similar mechanical properties. Thus, soft rock could be replaced with rock-like material in our investigation.

### 2.2. Testing Procedure

**Figure 4:**
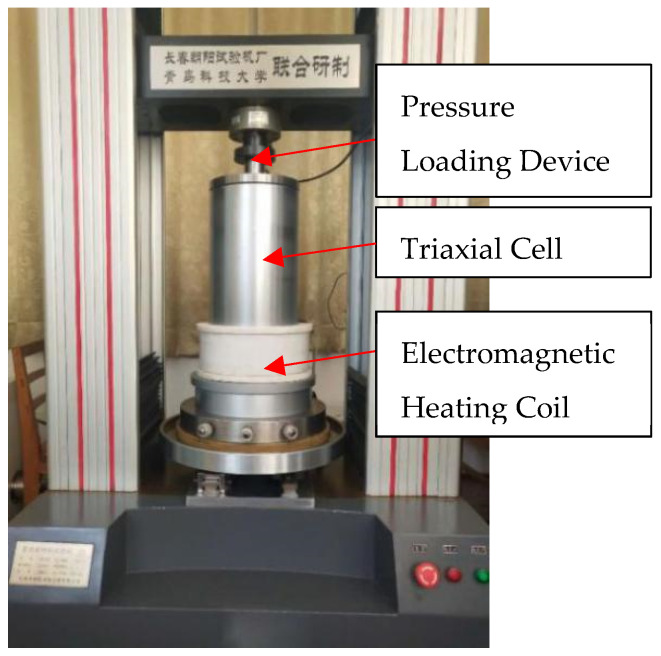
TAW-200 material mechanics testing machine.

In order to ensure that all specimens were at the same level of drying, all specimens were dried using an RPH-80 [28] temperature and humidity chamber from Qingdao University of Science and Technology before the compression test. After drying, compression tests were carried out at different temperatures and confining pressures using a TAW-2000 material mechanics testing machine (Figure 4). According to the ground temperature distribution in north China, the temperature in deep areas around 1000 m is 60 to 70 °C. Furthermore, the crustal stress value depends on the depth, whereby the horizontal crustal stress at a depth of 1000 m is around 19.35–33.29 MPa [29]. According to the similarity criterion, the confining pressure applied to the rock-like material should be lower than 8.32 MPa, such that the confining pressures in the test were 0, 1, 3, 5, and 7 MPa and the temperatures were 20, 30, 40, 50, and 60 °C. Each combination represented a test group, resulting in a total of 25 test groups in this study. Three tests were carried out for each test group, and the average values of peak stress and strain from the three tests were taken as the final result of this group. Furthermore, in order to investigate the influence of initial crack on the strength and deformation, contrast tests were carried out at 20–60 °C and 0 MPa with the intact specimen and crack specimen. In our previous investigation [30], we found that the crack with a 45° angle had the most significant influence on both strength and deformation. Thus, we chose 45° as the crack angle of the specimen.

**Figure 5:**
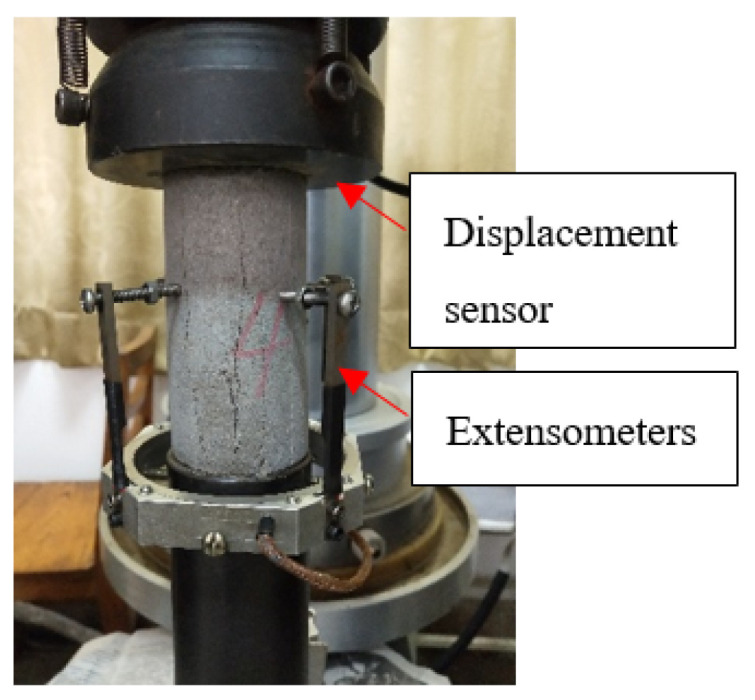
Extensometers and displacement sensor.

The test process was as follows: first, the triaxial cell was heated to a preset value at the heating rate of 2 °C/min using an electromagnet, which was maintained for 2 h to ensure a uniform temperature inside the specimen. The preset temperature was maintained throughout the test before applying confining pressures to the specimens. Finally, specimens were compressed using the TAW-2000 material mechanics testing machine at a loading rate of 50 N/s until specimens were damaged, thereby losing the capability of loading; stress–strain curves and deformation of the specimen were recorded at the same time. The radial deformation was measured by extensometers, and the axial deformation was measured by a displacement sensor, as exhibited in Figure 5.

## 3. Results and Discussion

### 3.1. Uniaxial Compression Test at Different Temperatures

**Figure 6:**
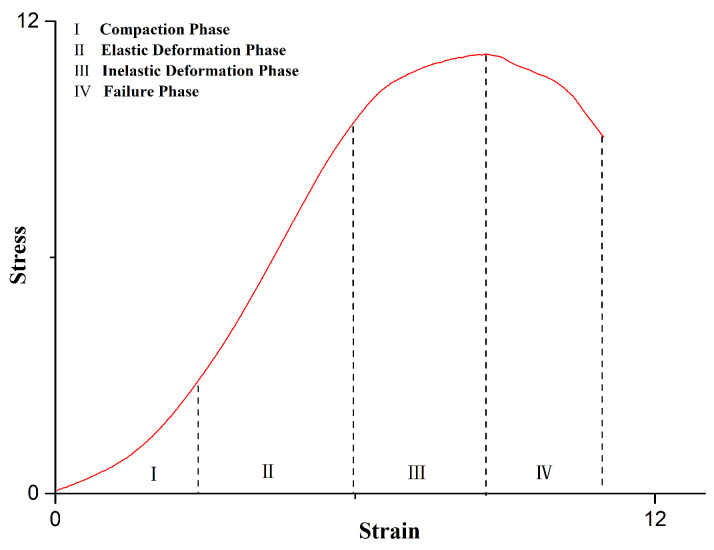
Four phases of the stress–strain curve for rock-like material.

**Figure 7:**
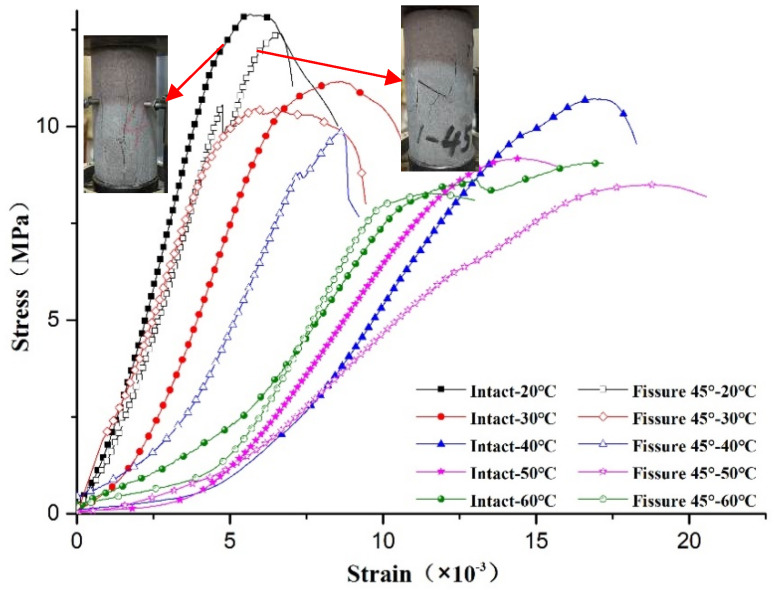
Stress–strain curves of uniaxial compression test.

The axial stress–strain curves of rock-like material at 20~60 °C were obtained via uniaxial compression tests. As shown in Figure 6, the stress–strain curves of rock-like material could be roughly divided into four phases: compaction phase, elastic deformation phase, inelastic deformation phase, and failure phase. Despite its nonobvious nature at low temperature (20 and 30 °C), the strain increased with temperature during the compaction phase. Due to the maldistribution of components and the difference in thermal expansivity of particles, thermal stress occurred during the period of heating. Thus, this phenomenon can be roughly attributed to the thermal damage of internal components resulting from asymmetrical thermal expansion, which resulted in an increase in strain during the compaction phase. We can see from Table 2 and Figure 7 that the strength of the intact specimen was higher than that of the cracked specimen at all test temperatures, and the strength decreased with temperature in both intact and cracked specimens. The main reason for the strength reduction in cracked specimens was the stress concentration at the crack tip [17]. We can see from Table 2 that the peak stress decreased with temperature in both intact and cracked specimens, with the minimum value appearing at 60 °C. As shown in the Figure 7, the cracks in the intact specimen were generated from the bottom of the specimen and expanded along the axial direction, whereas the new cracks in the injured specimen were generated from the crack tips and expanded perpendicularly to crack face.

### 3.2. Compression Test at Different Temperatures and Confining Pressures

#### 3.2.1. Influences of Temperature and Confining Pressure on Strength and Failure Mode

**Figure 8:**
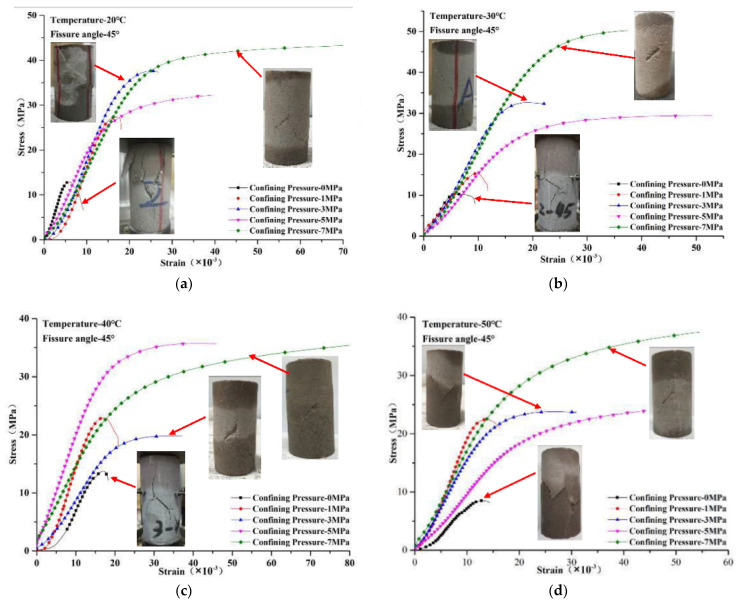

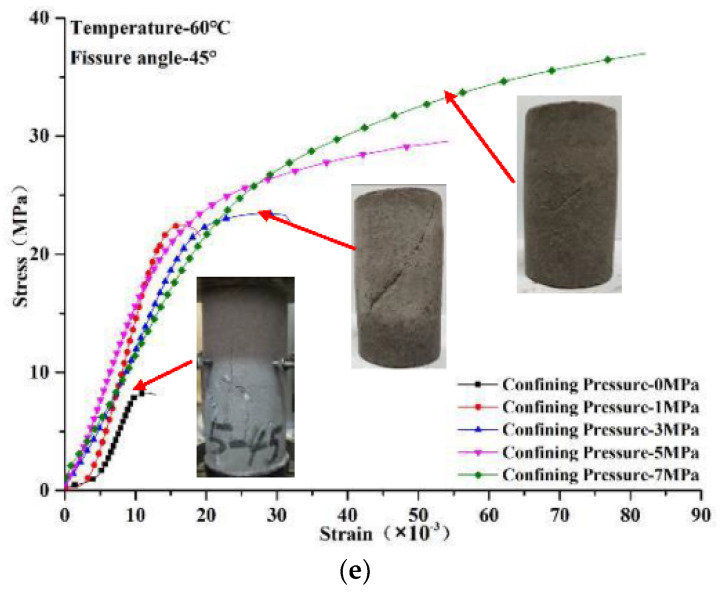
Stress–strain curves of compression test in different test conditions: (**a**) different confining pressure (temperature: 20 °C); (**b**) different confining pressure (temperature: 30 °C); (**c**) different confining pressure (temperature: 40 °C); (**d**) different confining pressure (temperature: 50 °C); (**e**) different confining pressure (temperature: 60 °C).

Failure mode compression tests were carried out on cracked specimens at different temperatures and confining pressures, and the stress–strain curves are shown in Figure 8. As shown in Figure 8, there were obvious peak stress points between the inelastic deformation phase and failure phase at the confining pressures of 0 and 1 MPa. The peak stress point was inconspicuous at the confining pressure of 3 MPa according to the stress–strain curves. However, there were no obvious peak stress points when the confining pressure was between 5 and 7 MPa, due to the failure mode of the rock-like material transforming to plastic failure with the increase in confining pressure. We can see from the failure specimen in Figure 8a that there were plenty of cracks on the surface of the specimen, and the failure mode is brittle failure under a confining pressure of 0.1 MPa. However, when the confining pressure was increased to 3 MPa, the number of cracks decreased and the size of the cracks was smaller than at low confining pressure. Moreover, there were no obvious cracks on the surface of the failure specimen, and strong deformation occurred in both axial and radial directions, which confirmed the transformation of the failure mode from brittle to plastic failure. It can be summarized that a smaller confining pressure led to more and bigger cracks on the surface of the failure specimen; a larger confining pressure led to fewer and smaller cracks on the surface of the failure specimen. The threshold of failure mode transformation was 3 MPa. A similar phenomenon was reported in Yang’s investigation of the failure mode of marble, which changed from brittle failure to plastic failure with the increase in confining pressure [6]. Accordingly, it is useless to confirm the specimen’s failure using the peak stress point in the stress–strain curve. As shown in Figure 7, the stress increased slowly after the inelastic deformation phase with confining pressure between 3 and 7 MPa. The criterion of specimen failure with high confining pressure was the occurrence of large deformation instead of the peak stress point.

It can be found by comparing Figure 8a–e that the temperature had less influence than the confining pressure on the failure mode of rock-like material. The duration in the inelastic deformation phase increased at confining pressures of 5 to 7 MPa, while the strain in the inelastic deformation phase increased with temperature. The main reason for increased strength can be attributed to the increase in non-deformability generated by temperature stress in the inelastic deformation phase.

**Figure 9:**
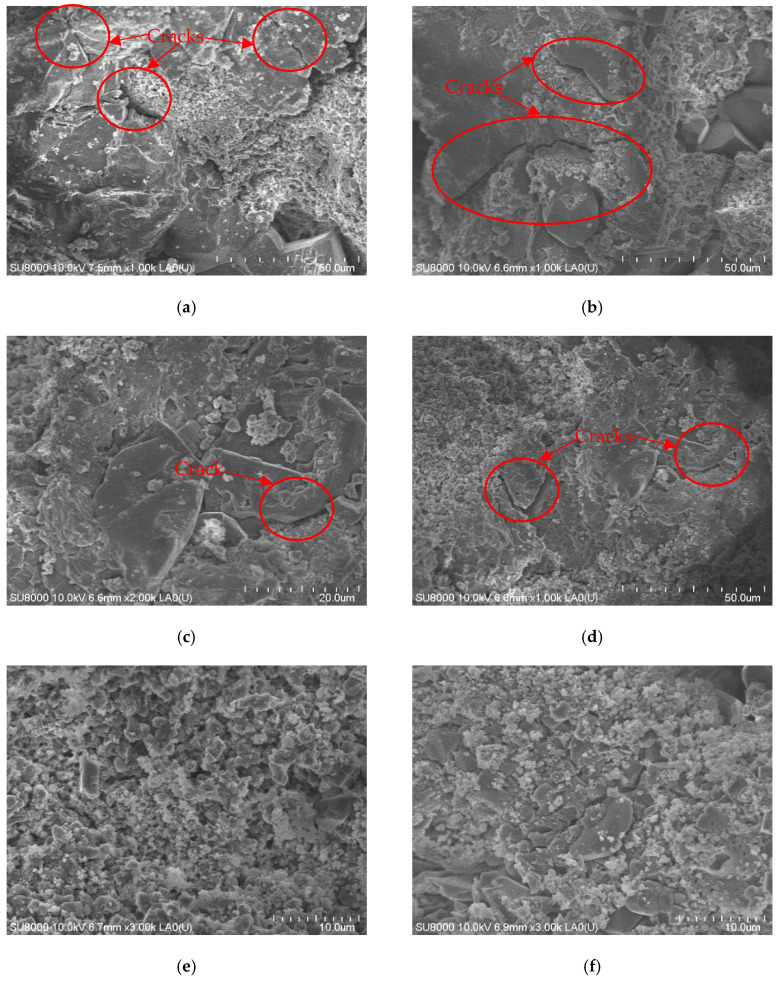
Fracture surface of the failure specimen. (**a**) Temperature 20 °C, confining pressure 0 MPa; (**b**) Temperature 60 °C, confining pressure 0 MPa; (**c**) Temperature 20 °C, confining pressure 3 MPa; (**d**) Temperature 60 °C, confining pressure 3 MPa; (**e**) Temperature 20 °C, confining pressure 7 MPa; (**f**) Temperature 60 °C, confining pressure 7 MPa.

According to the previous experiment results, 3 MPa is the critical stress value of brittle–plastic conversion. In order to explain the mechanism underlying how confining pressure affects the failure mode of rock-like material, we observed the fracture surface of the failure specimen via SEM (scanning electron microscopy). We can see from the pictures of the fracture surface that there were more and larger cracks on the surface at a confining pressure below 3 MPa. However, there were fewer and smaller cracks on the surface at a confining pressure above 3 MPa. For example, we can see from Figure 9a that there were more cracks on the fracture surface at a confining pressure of 0 MPa, and the cracks were larger compared to a confining pressure of 7 MPa. The number and size of the cracks decreased for confining pressures up to 3 MPa. Moreover, there were no obvious cracks on the fracture surface at a confining pressure of 7 MPa; instead, the component was compressed tightly due to the effects of confining pressure. This can be attributed to the effects of lateral stress (i.e., confining pressure), which led to the uncontrolled generation and propagation of cracks without lateral stress. Therefore, there were more cracks generated in the specimen at a confining pressure lower than 3 MPa, whereby the specimen failed due to crack propagation. Thus, the failure mode of the specimen was brittle at a low confining pressure. However, when the component was compressed tightly and the crack generation and propagation were inhibited due to the effect of confining pressure, the load capacity of the specimen was enhanced. The failure of the specimen was subsequently not due to the loss of load capacity but the large plastic deformation when the confining pressure exceeded the critical value. Thus, the failure mode of the specimen was plastic at a high confining pressure. Thus, the main reason for the brittle–plastic conversion was the effect of lateral stress, which suppressed the generation of new cracks and the propagation of initial cracks.

#### 3.2.2. Effect of Temperature and Confining Pressure on Rock-like Material Parameters

**Figure 10:**
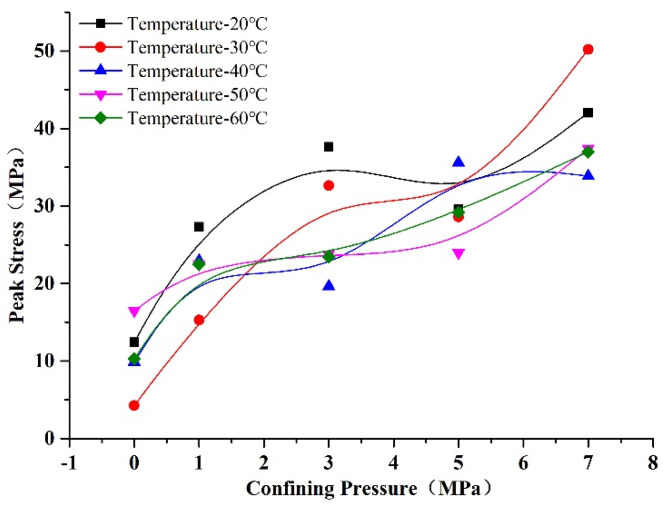
Changes in peak stress of rock-like material with confining pressure.

**Figure 11:**
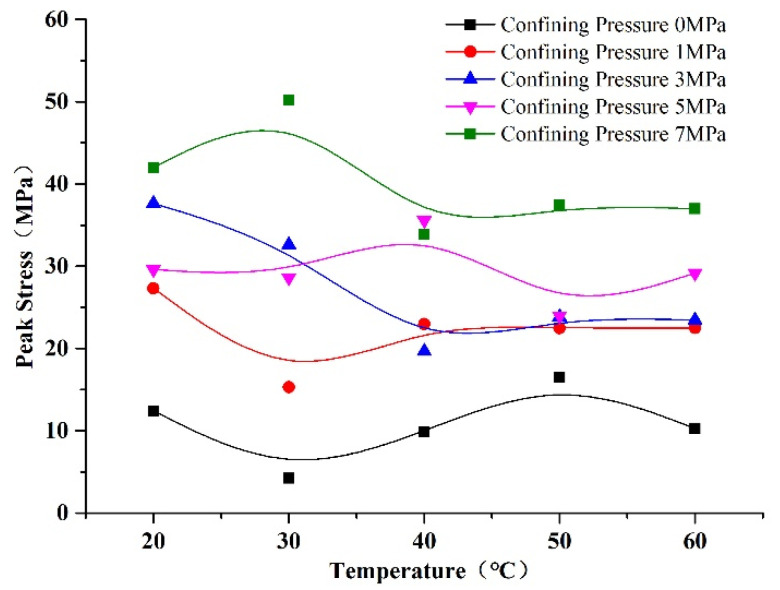
Changes in peak stress of rock-like material with temperature.

The relationships of peak stress with confining pressure and temperature are shown in Figure 10 and Figure 11. We can see from Figure 10 that the peak stress of rock-like material increased with confining pressure. We can infer that the initial cracks were sealed under the effect of increasing confining pressure; thus, the strength of the rock-like material was increased. We can see from Figure 11 that there was no obvious regularity of the peak stress under the effect of temperature at confining pressures of 0 and 1 MPa, whereby the peak stress fluctuated within a small range with temperature; on the other hand, the peak stress of the rock-like material decreased slowly with temperature at pressures of 3, 5, and 7 MPa. Temperature had less influence on the peak stress than confining pressure. The rock-like material peak stress decreased with temperature due to it being anisotropic, and the deformation of the internal microstructure was inhomogeneous with the effect of heating. Thus, new microcracks were generated, thereby decreasing the strength. However, in Zhao’s investigation, the peak stress of granite decreased obviously with temperature [31]. The reason for this difference is the temperature profile. In Zhao’s investigation, the specimens were heated first and then cooled to ambient temperature before the compression test, whereas, in this paper, the specimens were heated throughout the experiment. The strengthening effect of pyrogenic thermal stress disappears when specimens are cooled; thus, the effect of temperature on peak strain showed an obvious decrease in Zhao’s investigation.

**Figure 12:**
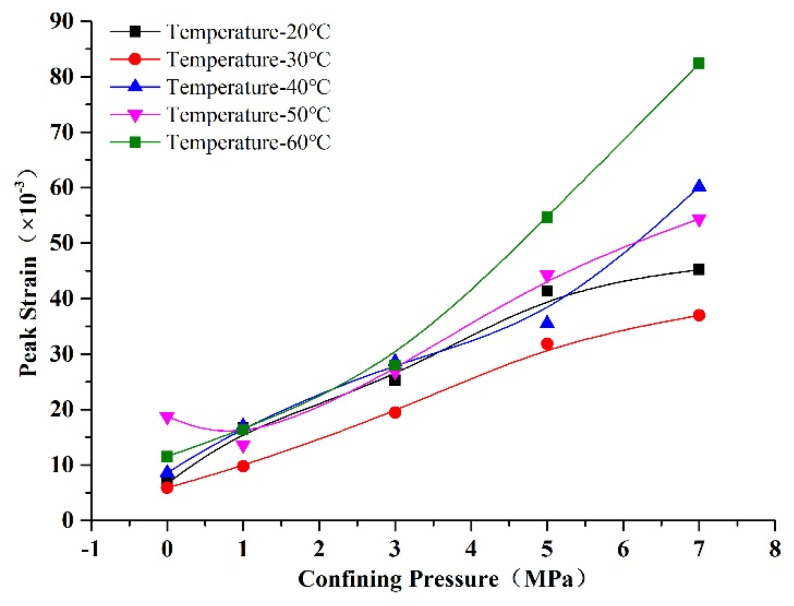
Changes in peak strain of rock-like material with confining pressure.

**Figure 13:**
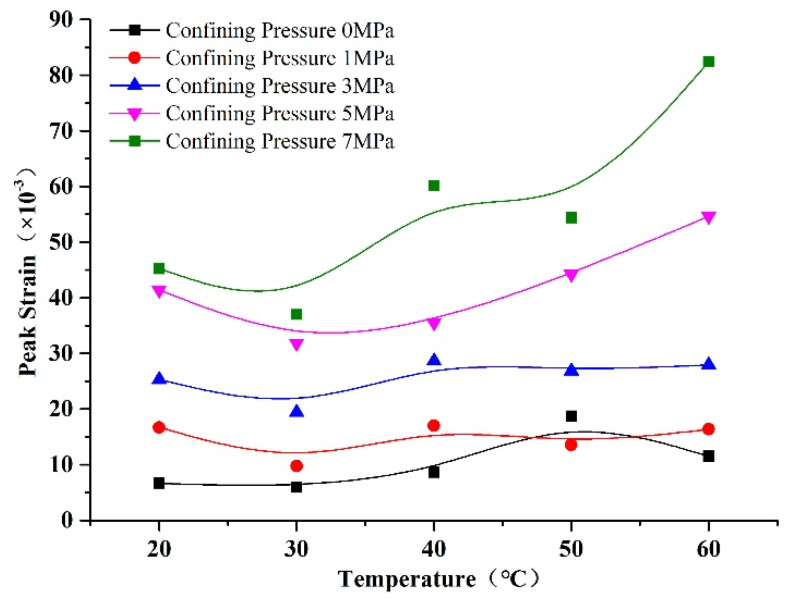
Changes in peak strain of rock-like material with temperature.

The relationships of peak strain with confining pressure and temperature are shown in Figure 12 and Figure 13. We can see from Figure 12 that the peak strain increased with confining pressure, due to the failure mode changing from brittle to plastic failure under the effect of confining pressure. Therefore, the strain in the inelastic deformation phase increased, and the total strain of the rock-like material increased accordingly [27]. We can see from Figure 13 that the strain increased with temperature, whereby the influence was weak at low confining pressure, but significant at high confining pressure. This was due to the coupled influence of temperature and confining pressure, which enhanced the deformation of the specimen. We can see from Figure 12 and Figure 13 that the influence of temperature on peak strain was smaller than that of confining pressure. The amplification of peak strain was small at confining pressures of 0, 1, and 3 MPa, whereas it was large at confining pressures of 5 and 7 MPa. This change tendency is coincident with granite deformation from Zhao’s investigation [31].

The influence of temperature on the rock-like material strength in the process of compression test was related to thermal stress and thermal damage. The components and the microcracks inside the specimen were expanded during heating, which led to them partially closing, thereby strengthening the specimen (i.e., thermal stress) [12]. The difference in expandability and distribution of internal components resulted in the production of new cracks during heating, thereby weakening the specimen (i.e., thermal damage) [13,14,15,16,17,18,19,20,21,22,23,24,25,26,27,28,29,30,31,32]. Thermal stress and thermal damage occur simultaneously during the heating process. When thermal stress is dominant, the effect of temperature on the specimen is positive, whereas, when thermal damage is dominant, the effect of temperature on the specimen is negative.

The Young’s modulus in this paper refers to the slope value of the elastic deformation phase in the stress–strain curves, according to Equation (1).

(1) $$E=\frac{\sigma_{t}-\sigma_{0}}{\varepsilon_{t}-\varepsilon_{0}}$$

where *E* is Young’s modulus (GPa), $\sigma_{0}$ is the stress at the initial point of the elastic deformation phase (MPa), $\sigma_{t}$ is the stress at the end of the elastic deformation phase (MPa), $\varepsilon_{0}$ is the strain at the initial point of the elastic deformation phase, and $\varepsilon_{t}$ is the strain at the end of the elastic deformation phase.

**Figure 14:**
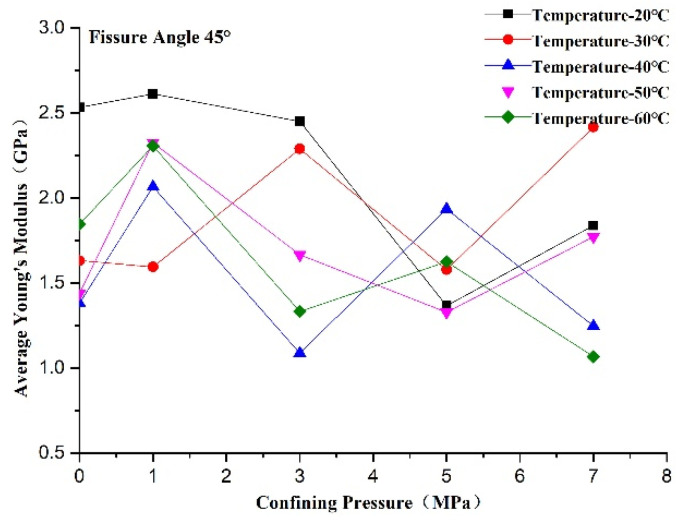
Changes in Young’s modulus of rock-like material with confining pressure.

**Figure 15:**
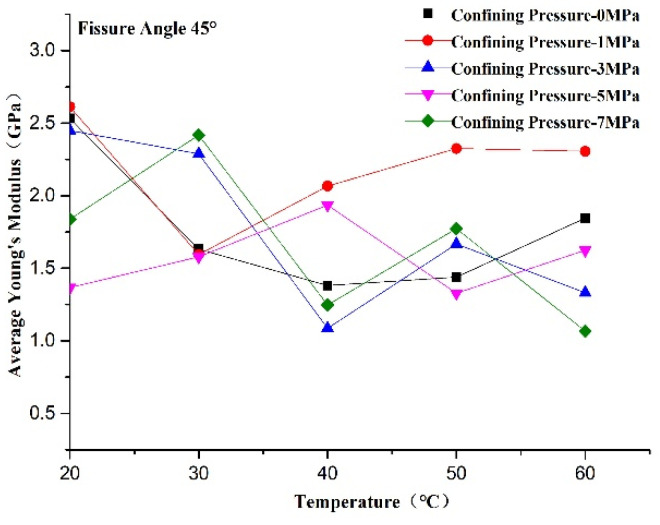
Changes in Young’s modulus of rock-like material with temperature.

The average Young’s modulus reflects the non-deformability of the rock-like material in the elastic deformation phase. A larger average Young’s modulus reflects stronger non-deformability of the rock-like material in the elastic deformation phase. We can see from Figure 14 that the average Young’s modulus increased first and then decreased with confining pressure when the temperature was constant. The maximum values of the average Young’s modulus appeared at a confining pressure of 1 MPa at all test temperatures except 30 °C. This suggests that the non-deformability of the rock-like material in the elastic deformation phase is lower at a confining pressure of 0 MPa and reaches its maximum value at 1 MPa. In addition, the failure mode changed from brittle to plastic failure with the increase in confining pressure, which resulted in an increase in strain in the elastic deformation phase. We can see from Figure 15 that the influence of temperature on Young’s modulus was inconspicuous at confining pressures of 1 and 5 MPa, whereas it decreased with temperature at confining pressures of 0, 3, and 7 MPa. This is attributed to thermal damage dominating during heating and new micro-damage being generated, whereby the non-deformability in the elastic deformation phase decreased due to the generation of new cracks, which enhanced the damage degree of the specimen.

The behavior of Young’s modulus was in accordance with Morteza’s results [8] but in contrast to Yang’s results [6]. This can be attributed to the strength difference of the specimens, whereby the specimens in Yang’s test were hard and, thus, had increased strength. Accordingly, the non-deformability in the elastic deformation phase was higher in Yang’s test. However, the specimen was soft rock-like material in this study, exhibiting lower strength; thus, the applied confining pressure was close to its uniaxial compressive strength. Therefore, new cracks were more likely to be produced, resulting in a reduction in Young’s modulus.

## 4. Conclusions

A laboratory study was conducted to investigate the influence of initial crack, temperature, and confining pressure on the deformation and strength characteristics of rock-like material, which has important reference value for revealing the deformation and strength characteristics of deep rock mass. The conclusions are summarized as follows:

(1) Compared with the intact specimen, the strength of the rock-like material with initial crack decreased due to the stress concentration around the tips of the initial crack, which led to the generation of more microcracks around the tips. As observed from the surface of the failure specimen, we can infer that the existence of the initial crack generated new microcracks compared with the intact specimen.
(2) Thermal stress was generated due to the increase in temperature, thereby decreasing the strength and increasing the deformation of the shale-like specimen. This can be attributed to the generation of microcracks inside the specimen, which decreased its compressive capacity. Meanwhile, the deformation of the specimen increased due to the generation of microcracks.
(3) The deformation of the rock-like material was affected by both temperature and confining pressure, but confining pressure was the dominant factor. The failure mode of the rock-like material changed with confining pressure. The failure mode was brittle failure at confining pressures of 0 and 1 MPa, whereas it was plastic failure at confining pressures of 5 and 7 MPa. A confining pressure of 3 MPa was the critical value for the transition from brittle to plastic failure. The influence of temperature on the rock-like material was mainly reflected in the later phase of inelastic deformation under confining pressures of 5 and 7 MPa, leading to an increase in deformation with temperature.
(4) Both strength and deformation were affected by the combined influence of temperature, confining pressure, and initial damage. However, the degree of influence and the mechanism of each factor was different. Temperature decreased the strength and increased the deformation due to the generation of thermal stress. Confining pressure enhanced the strength due to lateral stress restricting the deformation and increased the deformation due to the change in failure mode. Initial crack decreased the strength and exaggerated the deformation due to the stress concentration generating more cracks around the initial tips. Confining pressure and initial crack had a significant influence on the strength and deformation, whereas the influence of temperature was minor.

## 5. Limitations and Future Works

In this paper, the mechanical properties of crack specimens under the coupled effect of temperature and confining pressure were studied, and a preliminary exploration of mechanical properties of injured rock mass under various conditions was achieved. However, due to the cracks in the specimen having a specific angle, the influence of the internal damage of rock mass on its mechanical properties could not be fully reflected. Therefore, in future work, it is necessary to investigate the mechanical properties of randomly damaged specimens.

## Figures and Tables

**Table 1:** Comparison table of parameters between rock-like material and rock.

	Parameters	Density (g/cm^3^)	Young’s Modulus (GPa)	Compressive Strength (MPa)	Internal Friction Angle (°)	Cohesion Force (MPa)
Class						
Parameters of Rock		1.7–2.5	1.0–3.3	10–25	27–60	3.43–46.6
Parameters of Rock-Like Material ^a^		1.902	1.864	12.077	32.751	4.199

Note: ^a^ Parameters of rock-like material are the average values from the intact rock-like material’s testing results.

**Table 2:** Comparison of rock-like material’s peak stress at different temperatures.

Specimen Type	20 °C	30 °C	40 °C	50 °C	60 °C
	Peak Stress (MPa)				
Intact Specimen	12.906	11.167	10.716	9.164	9.048
Cracked Specimen *	12.409	10.464	9.853	8.497	8.279

* The value of peak stress is the average value of three experiments.

## Data Availability

The data used to support the findings of this study are available from the corresponding author upon request.
